# Post-ozonation in a municipal wastewater treatment plant improves water quality in the receiving stream

**DOI:** 10.1186/s12302-015-0068-z

**Published:** 2016-01-05

**Authors:** Roman Ashauer

**Affiliations:** Environment Department, University of York, Heslington, York, YO10 5DD UK

**Keywords:** Treatment of wastewater, Good biological status, Stream macroinvertebrates, Trait-based ecological risk assessment, Micropollutant removal, Biodiversity

## Abstract

**Background:**

Removal of organic micropollutants from wastewater by post-ozonation has been investigated in a municipal wastewater treatment plant (WWTP) temporarily upgraded with full-scale ozonation, followed by sand filtration, as an additional treatment step of the secondary effluent. Here, the SPEAR (species at risk) indicator was used to analyse macroinvertebrate abundance data that were collected in the receiving stream before, during and after ozonation to investigate whether ozonation improved the water quality.

**Results:**

The SPEAR values indicate a better water quality downstream the WWTP during ozonation. With ozonation the relative abundance of vulnerable macroinvertebrates in the stream receiving the treated wastewater increases from 18 % (CI 15–21 %) to 30 % (CI 28–32 %). This increase of 12 % (CI 8–16 %) indicates improved ecological quality of the stream and shifts classification according to the Water Framework Directive from poor to moderate.

**Conclusions:**

The SPEAR concept, originally developed to indicate pesticide stress, also appears to indicate toxic stress by a mixture of various micropollutants including pharmaceuticals, personal care products and pesticides. The responsiveness of the SPEAR indicator means that those macroinvertebrates that are vulnerable to pesticide pollution are also vulnerable to micropollutants from WWTPs. The change in the macroinvertebrate community downstream the WWTP indicates that toxicity by pollutants decreased by more than one order of magnitude during ozonation. Ozonation followed by sand filtration has favourable impacts on the composition of the macroinvertebrate community and can improve the water quality in the receiving stream.

**Electronic supplementary material:**

The online version of this article (doi:10.1186/s12302-015-0068-z) contains supplementary material, which is available to authorized users.

## Background

Micropollutants, for example, pharmaceuticals, personal care products or biocides, are discharged with municipal wastewater and may be hazardous to the environment [1–3]. Ozonation is one of the techniques suggested for tertiary treatment to remove micropollutants from wastewater [1, 4, 5], but the ecotoxicological consequences of wastewater ozonation are ambiguous [6]. Formation of toxic by-products through ozonation is possible, although these can be eliminated in subsequent sand filtration [7, 8].

Removal of organic micropollutants from wastewater by post-ozonation has recently been investigated in a municipal wastewater treatment plant [9, 10]. The wastewater treatment plant (WWTP) Wüeri in Regensdorf, Switzerland was upgraded with ozonation as an additional treatment step of the secondary effluent. Ozonation followed by sand filtration was shown to remove most of the micropollutants [9]. Of those compounds that were detected in the secondary effluent, 17 compounds were reduced by more than 90 % during ozonation, another 17 compounds between 50 and 90 % and four compounds were reduced by less than 50 % [9]. A complementary study using an in vitro mode-of-action-based bioassay battery also demonstrated that ozonation reduced the toxicity of the mixture of micropollutants in the effluent in this experiment [11]. The bioassay battery used enriched samples and measured mode-of-action specific toxicity. The treatment efficiency of the ozonation step was 65 and 76 % for non-specific toxicity in the bacterium *Vibrio fischeri* and the algae *Pseudokirchneriella subcapitata*, respectively, 86 % for inhibition of photosystem II in algae, 86 % for estrogenicity, 60 % for inhibition of acetylcholinesterase and complete removal of genotoxicity [11]. Consistent with chemical analysis, micropollutants which are readily oxidised by ozonation, e.g. those causing estrogenicity, showed greatest reduction of toxicity [11]. Furthermore, another complementary study using fish early life stage toxicity tests (FELST) [8] found that the ozonation step led to reduced growth and development in the FELST, although post-treatment with sand filtration eliminated such toxic effects. Altogether these three studies showed reduced micropollutant loads and toxicity in the wastewater after ozonation together with sand filtration compared to conventionally treated wastewater.

The ultimate aim of upgrading WWTPs, for example, with ozonation followed by sand filtration, is to improve the water quality in the receiving stream. Hence, I investigated if ozonation of wastewater improved the water quality in the Furtbach, the stream into which the WWTP in Regensdorf discharges its effluent. My objective is to use the macroinvertebrate data that were collected as part of the full-scale ozonation experiment [8, 9, 11] and investigate whether ozonation followed by sand filtration improved the water quality as indicated by the abundance of vulnerable macroinvertebrates. The macroinvertebrate data were analysed using the SPEAR (species at risk) indicator. Specifically, I asked: How much did the proportion of vulnerable species in the receiving stream’s macroinvertebrate community change, when the wastewater was ozonated?

## Results and discussion

**Fig. 1:**
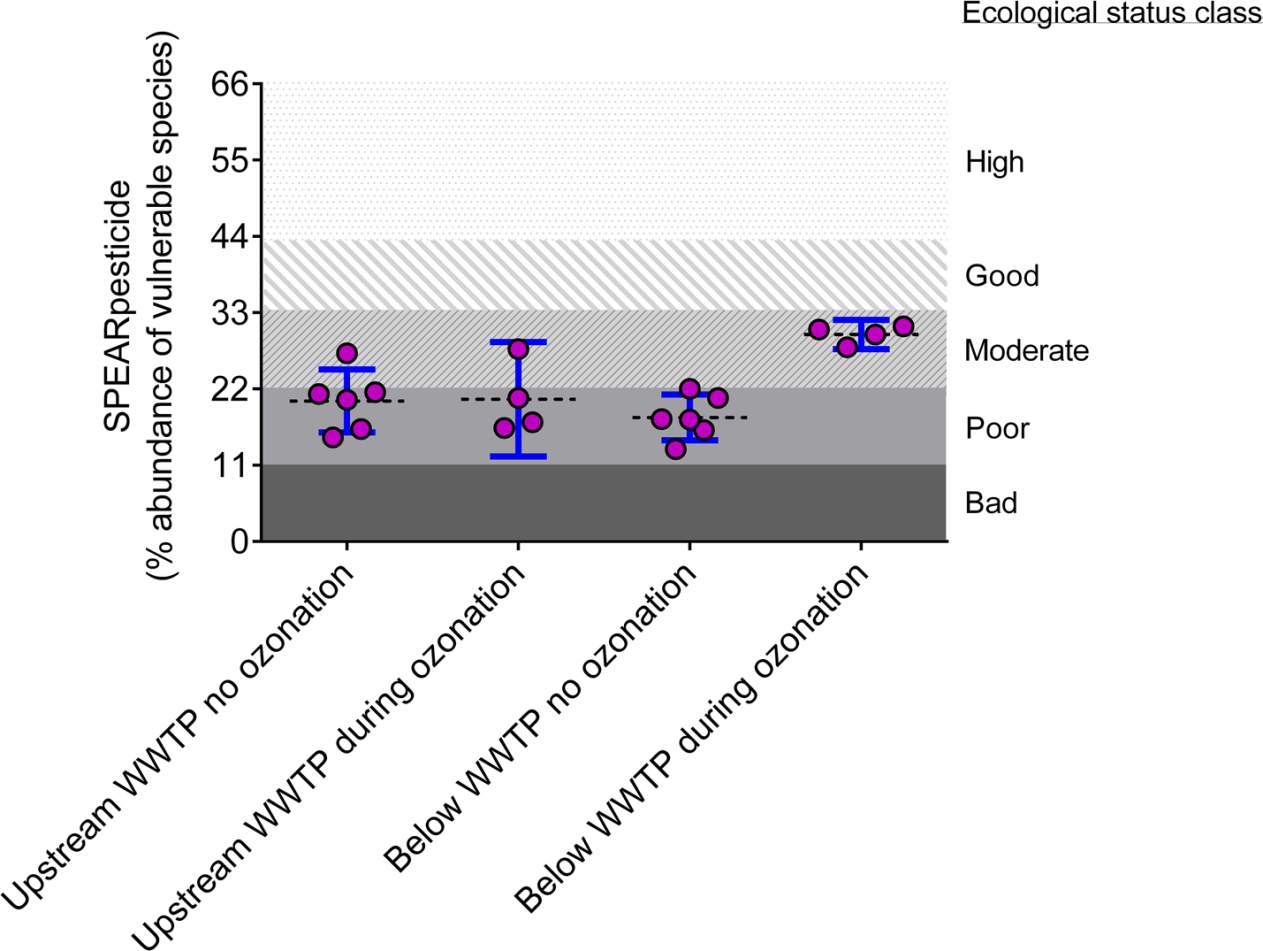
Relative abundance of vulnerable taxa. SPEAR indicates the fraction of vulnerable species in the stream with and without ozonation followed by sand filtration (*circles* macroinvertebrate surveys, *dotted line* mean, *blue bars* 95 % confidence intervals). Ozonation increases the abundance of vulnerable species from 18 to 30 %. This increase of 12 % (CI 8–16 %) indicates improved ecological quality of the stream and shifts classification according to the WFD from poor to moderate [12]

According to the classification of Beketov et al. [12] the macroinvertebrates indicate a poor biological status of the stream upstream and downstream of the WWTP without ozonation (Fig. 1). The poor quality of the upstream sites can be, at least partially, explained by pollution upstream of the WWTP [13]. Ozonation increases the abundance of vulnerable macroinvertebrates in the stream receiving the treated wastewater from 18 % (CI 15–21 %) to 30 % (CI 28–32 %). This increase of 12 % (CI 8–16 %) indicates improved ecological quality of the stream and shifts classification according to the WFD from poor to moderate [12].

Other researchers have found that WWTP effluents change the assemblages of macroinvertebrates in receiving stream mesocosms [14], although they attributed the effect to increased nutrients and reduced dissolved oxygen. Here, ozonation followed by sand filtration increases the relative abundance of vulnerable species present downstream of the WWTP and leads to an improved water quality classification. Ozonation even appears to improve the water quality downstream of the WWTP compared to upstream (Fig. 1). This seems plausible given the large relative volume that the wastewater contributes to the stream, although the low replication within this study and the large number of possible confounding factors requires further research on this aspect.

It is noteworthy that the effect of the ozonation treatment can be detected in the stream macroinvertebrate composition after only 8 and 16 months. The number of SPEAR_pesticides_ values in each group was small and consisted of data from different locations and sampling dates (spring and autumn), all of which can be assumed to increase variability in the macroinvertebrate community composition. The raw data of this analysis, i.e. taxa lists and abundances, are given in the Additional file 1.

Another finding is that the SPEAR concept, originally developed to indicate pesticide stress, also appears to indicate toxic stress by a mixture of various micropollutants including pharmaceuticals, personal care products and pesticides. The responsiveness of the SPEAR indicator, also known as SPEAR_pesticides_, does not necessarily mean that the stressors are pesticides; rather it means that those macroinvertebrates that are vulnerable to pesticide pollution are also vulnerable to pollution by micropollutants from WWTPs. An improvement of the ecological status in the receiving stream due to the additional ozonation step followed by sand filtration as indicated by SPEAR is consistent with the reduction of overall micropollutant load found by chemical analysis [9] and monitoring with bioassays [11]. A differentiation of the effects of various micropollutants was not possible with SPEAR. Chemical analysis of the receiving stream water before and during ozonation also confirmed the reduction of micropollutant loads, for example, the concentrations of carbamazepine, diclofenac, clarithromycin and sulfamethoxazole were reduced by ozonation from 0.51 μg/L to below 3 ng/L, 0.41 μg/L to below 10 ng/L, 0.12 μg/L to below 3 ng/L and 0.12 μg/L to below 6 ng/L, respectively [9, 15]. Furthermore, feeding trials with leaf discs conditioned in wastewater from the same WWTP as studied here showed that *Gammarus fossarum* preferred the leaf discs that were conditioned in wastewater treated with high doses of ozone over those leaf discs that were conditioned in untreated wastewater [6] and in situ feeding rate trials showed that ozonation increases detritus processing in the stream [16].

Analysis of the macroinvertebrate community on the receiving water bodies downstream of WWTPs can clearly contribute to answer the question whether post-treatment technologies help achieve water quality goals, in particular when existing knowledge about vulnerability of species is built into the data analysis as ,for example, with the SPEAR concept. Not all WWTPs contribute as much water volume to the stream as the one studied here, thus future studies may need to increase their power by larger sample size and improved design [17, 18].

The impact of WWTP effluents on stream macroinvertebrate assemblages has been documented before [19, 20]. The clear effects of upgrading the WWTP with ozonation, more specifically the increase in vulnerable species in downstream samples from 18 to 30 %, would correspond to a reduction of toxicant loads by approximately 1.5 toxic units (*Daphnia magna*) according to the regressions in [21]. In other words the change in SPEAR in the macroinvertebrate community downstream the WWTP indicates that toxicity by pollutants decreased by more than one order of magnitude during ozonation.

## Conclusion

The previously reported reduction in chemical loads and reduced toxicity measured by an in vitro bio-test battery during ozonation followed by sand filtration in the WWTP has favourable impacts on the composition of the macroinvertebrate community and water quality in the receiving stream.

## Methods

### Study site

The WWTP discharges into a small stream (Furtbach) with a catchment of 12 km^2^ consisting of 24 % forest, 42 % agriculture and 29 % urban use (5 % other uses). The Furtbach stream has an average slope of 0.1 %, holds water all year round and the substrate consists mostly of large (fist to nut size) to small (nut to pea size) gravel with 10–20 % sand and 10 % or less silt at all four macroinvertebrate sampling sites [22]. The Furtbach originates from a small lake approximately 5 km upstream of the WWTP and discharges into the river Limmat approximately 9 km downstream. More details about the sampling site characteristics can be found in [22].

The WWTP approximately doubles the discharge in the stream (WWTP treats 5500 m^3^ d^−1^ on average under dry conditions, WWTP discharge ranges from 30 to 120 L s^−1^ and constitutes ca. 60 % of the water in the stream under dry weather conditions [9]). The WWTP consists of primary sedimentation, activated sludge treatment (nitrifying and denitrifying) and secondary clarification followed by sand filtration. The ozonation step was added after the secondary clarifier and before the sand filtration. The ozonation reactor had a retention time of 8–15 min during dry conditions and 3 min during storm water (which was judged not sufficient under stormwater conditions [9]). Ozonation followed by sand filtration was in operation, with short breaks, from July 2007 until the end of October 2008 [15]. Then the ozonation equipment was removed from the WWTP. Ozone dose and residence time in the reactor varied between 357 and 1157 g_O3_/kg_DOC_ and 4–10 min, respectively [11] and were regulated based on online measurements of dissolved organic carbon. More details on the treatment processes, chemistry of the wastewater and operation of the ozonation can be found in [9] and [15], including a wide range of additional parameters measured.

**Fig. 2:**
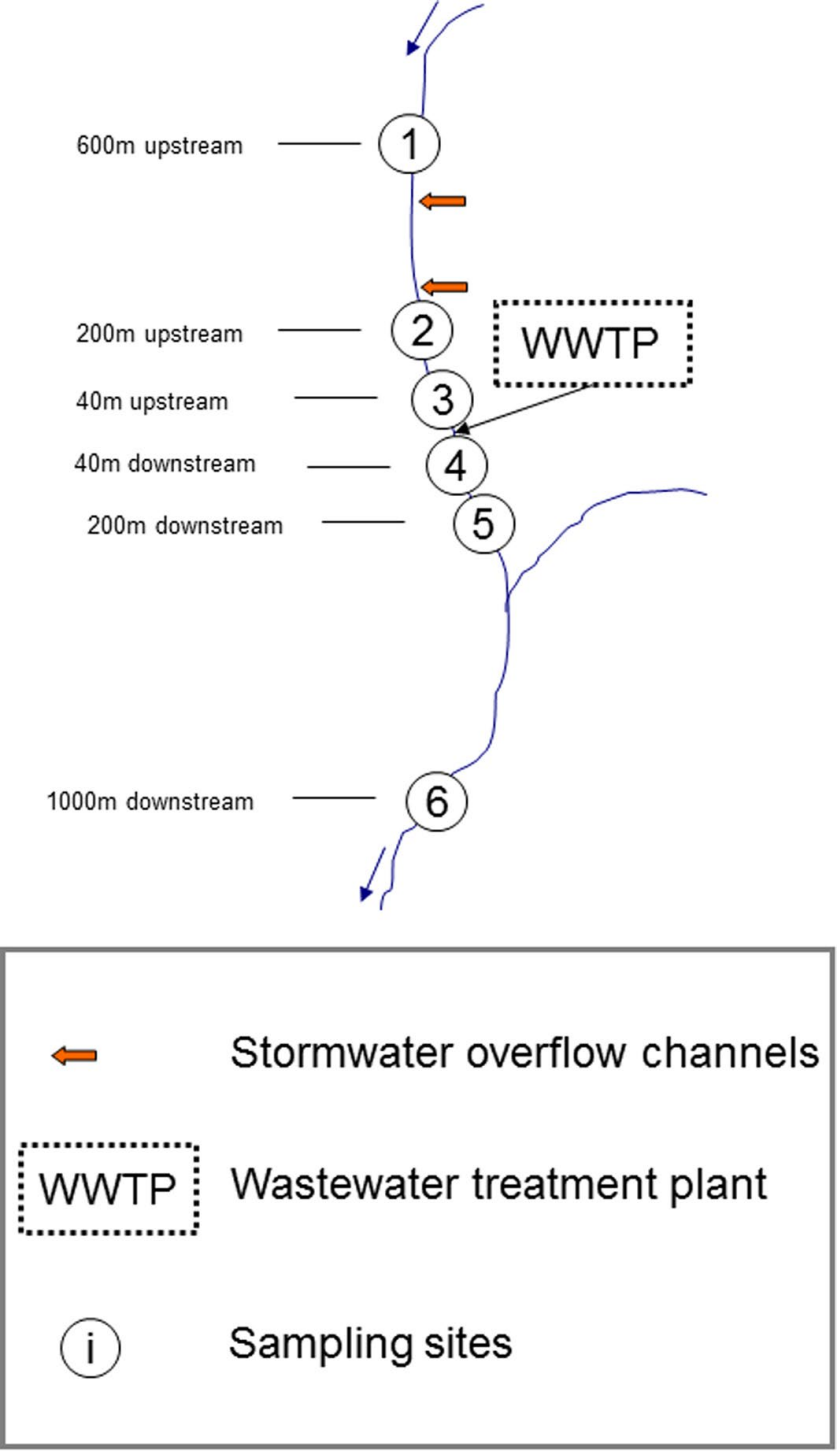
Location of the WWTP and the sampling sites at the stream Furtbach

Various sources of pollution upstream of the WWTP exist, for example, several storm water overflow channels discharge in the stream (Fig. 2), in June 2007 there was a contamination incident with an unspecified fungicide [15] and chemical analysis of the upstream water in June 2007 found several pesticides or biocides and their biotransformation products, as well as some pharmaceuticals in the ng/L range [13].

### Macroinvertebrate data

There were three sampling sites upstream and three downstream of the WWTP (see Table 1; Fig. 2). Before, during and after ozonation followed by sand filtration was installed at the wastewater treatment plant, the macroinvertebrates in the receiving stream were sampled, identified to species or family level (according to [23]) and abundances recorded [22]. There were two sampling dates before (5 October 2006, 26 February 2007), two during the period with ozonation followed by sand filtration (26 February 2008, 20 October 2008) and two sampling dates after the ozonation treatment was dismantled (10 March 2014, 14 October 2014). Thus, the ozonation was in operation already for 8 and 16 months when the macroinvertebrates were sampled to measure effects of the ozonation treatment in 2008 and the ozonation treatment had been dismantled for over 5 years before the sampling in 2014. The 2006–2008 macroinvertebrate data were collected by AquaPlus, Zug, Switzerland [22] on behalf of AWEL (Amt für Abfall, Wasser, Energie und Luft; Zürich, Switzerland). The macroinvertebrate data from the year 2014 were provided by AWEL (Patrick Steinmann, pers. comm.). More details and raw data can be found in [15, 22], as well as on the website of AWEL (http://www.gewaesserqualitaet.zh.ch).

**Table 1:** Sampling sites, dates and SPEAR values

Ozonation	Sampling date	Sampling site (site number in brackets, see Fig. 1)	Coordinates^a^	SPEAR (% relative abundance of species at risk)^b^
Group A				
No	5 October 2006	600 m upstream of WWTP (1)	2′676′819/1′256′070	16.20
No	5 October 2006	200 m upstream of WWTP (2)	2′676′457/1′256′202	21.27
No	26 February 2007	600 m upstream of WWTP (1)	2′676′098/1′256′159	21.52
No	26 February 2007	200 m upstream of WWTP (2)	2′675′322/1′256′133	27.15
No	10 March 2014	40 m upstream of WWTP (3)	2′676′296/1′256′225	20.42
No	14 October 2014	40 m upstream of WWTP (3)	2′676′296/1′256′225	15.03
Mean (95 % confidence intervals)				20.27 (15.72, 24.81)
Group B				
Yes	20 October 2008	600 m upstream of WWTP (1)	2′676′819/1′256′070	16.38
Yes	20 October 2008	200 m upstream of WWTP (2)	2′676′457/1′256′202	27.75
Yes	26 February 2008	600 m upstream of WWTP (1)	2′676′098/1′256′159	17.20
Yes	26 February 2008	200 m upstream of WWTP (2)	2′675′322/1′256′133	20.70
Mean (95 % confidence intervals)				20.51 (12.27, 28.75)
Group C				
No	5 October 2006	200 m downstream of WWTP (5)	2′676′819/1′256′070	13.31
No	5 October 2006	1000 m downstream of WWTP (6)	2′676′457/1′256′202	20.71
No	26 February 2007	200 m downstream of WWTP (5)	2′676′098/1′256′159	16.07
No	26 February 2007	1000 m downstream of WWTP (6)	2′675′322/1′256′133	17.58
No	10 March 2014	40 m downstream of WWTP (4)	2′676′211/1′256′205	22.03
No	14 October 2014	40 m downstream of WWTP (4)	2′676′211/1′256′205	17.64
Mean (95 % confidence intervals)				17.89 (14.59, 21.19)
Group D				
Yes	20 October 2008	200 m downstream of WWTP (5)	2′676′819/1′256′070	30.57
Yes	20 October 2008	1000 m downstream of WWTP (6)	2′676′457/1′256′202	29.84
Yes	26 February 2008	200 m downstream of WWTP (5)	2′676′098/1′256′159	31.01
Yes	26 February 2008	1000 m downstream of WWTP (6)	2′675′322/1′256′133	28.01
Mean (95 % confidence intervals)				29.86 (27.75, 31.96)

^a^Geographic coordinates: North/East

^b^See Eq. (1), indicator also known as SPEAR_pesticides_

### The SPEAR indicator and micropollutants

The SPEAR concept was developed as a tool to reveal impacts on stream macroinvertebrate communities related to chemical stress by pesticides [21, 24]. Species are classified according to their vulnerability into species at risk and species not at risk. Vulnerability classification takes into account ecological and physiological traits of the species, more specifically the toxicological sensitivity to organic pollutants including pesticides [25] as the only physiological trait, as well as the generation time, migration ability and time of emergence as ecological traits [21, 24]. Although some methodical aspects of the SPEAR approach such as the sensitivity ranking relative to *D. magna* and the neglect of mode-of-action specific sensitivity differences can be criticised [26], SPEAR values were shown to correlate with pesticide contamination in several catchments throughout Europe [24, 27], also when family-level data were used [12]. The approach taken here, using the SPEAR concept, assumes that the species traits that make SPEAR indicative of chemical stress by pesticides are also defining the vulnerability of macroinvertebrates to micropollutants present in WWTP effluent.

### Calculation of species at risk (SPEAR)

The 2006 to 2008 taxa lists and their abundance [22] were entered into the SPEAR web calculator (http://www.systemecology.eu/SPEAR/index.php, accessed 7 June 2010), whereas the 2014 macroinvertebrate data were analysed using the SPEAR calculator v0.9.0 (http://www.systemecology.eu/spearcalc/, accessed 6 November 2015, using family-level taxa, default traits and no recovery areas). Four taxa [Ostracoda (1 entry), Collembola (3 entries), *Gordius aquaticus* (4 entries), Podura (1 entry)] were deleted because they could not be found in the SPEAR database. In the web calculator Central Europe was selected as region, SPEAR_pesticides_ was calculated and absence or presence of recovery areas was not assessed (no values assigned). The explanatory power of the SPEAR indicator does not suffer significantly if family-level data are used instead of species-level data [12], the taxonomic resolution of the macroinvertebrate data used here is sufficient.

The relative abundance of species at risk (vulnerable species) is calculated as [27]

1 $$\%\, SPEAR = \frac{{\sum\limits_{i = 1}^{n} {\log (x_{i} + 1) \times y} }}{{\sum\limits_{i = 1}^{n} {\log (x_{i} + 1)} }},$$

where *n* is the number of taxa, *x*_*i*_ is the abundance of taxon *i* and *y* is 1 if the taxon *i* is classified as species at risk (vulnerable), otherwise *y* is 0.

### Statistical analysis

The SPEAR data can be grouped into four groups A, B, C and D (Table 1, Fig. 1) to better illustrate the analysis. These groups are (A) without ozonation upstream the WWTP, (B) during ozonation upstream the WWTP, (C) without ozonation below the WWTP and (D) with ozonation below the WWTP (Table 1). As the macroinvertebrate samples consist of only few replicates I followed recent developments in statistical reasoning and calculated the confidence interval (CI) of the difference that ozonation makes [28, 29]. Thus, I answered one question with the analysis of the SPEAR data: How much of a difference did ozonation make for the proportion of vulnerable species in the macroinvertebrate community in the receiving stream? The difference was calculated assuming normally distributed errors and equal variances and was carried out using GraphPad Prism version 6.03 (http://www.graphpad.com). All CIs are 95 % confidence intervals.

My analysis assumes that there is no effect of season and distance to the WWTP in the SPEAR data. Alternatively one can carry out a paired analysis, which reduces the number of data points to four in each group and results in larger confidence intervals. However, the results are very similar to those given above: ozonation increases the abundance of vulnerable macroinvertebrates downstream the WWTP from 17 % (CI 12–22 %) to 30 % (CI 28–32 %). This increase of 13 % (CI 7–19 %) also shifts classification according to the WFD from poor to moderate [12].

## Additional file

10.1186/s12302-015-0068-z Lists of taxa and abundances in each macroinvertebrate sample are available on-line.

## Abbreviations

WWTP: wastewater treatment plant
SPEAR: species at risk (SPEAR_pesticides_)
FELST: fish early life stage toxicity test
AWEL: Amt für Abfall, Wasser, Energie und Luft; Zürich, Switzerland (local authority)

## References

[1] Ternes T (2007). The occurrence of micopollutants in the aquatic environment: a new challenge for water management. Water Sci Technol.

[2] Schwarzenbach RP, Escher BI, Fenner K, Hofstetter TB, Johnson CA, von Gunten U (2006). The challenge of micropollutants in aquatic systems. Science.

[3] Ternes TA, Joss A, Siegrist H (2004). Scrutinizing pharmaceuticals and personal care products in wastewater treatment. Environ Sci Technol.

[4] Paraskeva P, Graham NJD (2002). Ozonation of municipal wastewater effluents. Water Environ Res.

[5] Huber MM, Göbel A, Joss A, Hermann N, Löffler D, McArdell CS (2005). Oxidation of pharmaceuticals during ozonation of municipal wastewater effluents: a pilot study. Environ Sci Technol.

[6] Bundschuh M, Gessner MO, Fink G, Ternes TA, Sögding C, Schulz R (2011). Ecotoxicological evaluation of wastewater ozonation based on detritus-detritivore interactions. Chemosphere.

[7] Stalter D, Magdeburg A, Oehlmann J (2010). Comparative toxicity assessment of ozone and activated carbon treated sewage effluents using an in vivo test battery. Water Res.

[8] Stalter D, Magdeburg A, Weil M, Knacker T, Oehlmann J (2010). Toxication or detoxication? In vivo toxicity assessment of ozonation as advanced wastewater treatment with the rainbow trout. Water Res.

[9] Hollender J, Zimmermann SG, Koepke S, Krauss M, McArdell CS, Ort C (2009). Elimination of organic micropollutants in a municipal wastewater treatment plant upgraded with a full-scale post-ozonation followed by sand filtration. Environ Sci Technol.

[10] Zimmermann SG, Wittenwiler M, Hollender J, Krauss M, Ort C, Siegrist H (2011). Kinetic assessment and modeling of an ozonation step for full-scale municipal wastewater treatment: micropollutant oxidation, by-product formation and disinfection. Water Res.

[11] Escher BI, Bramaz N, Ort C (2009). JEM Spotlight: monitoring the treatment efficiency of a full scale ozonation on a sewage treatment plant with a mode-of-action based test battery. J Environ Monit.

[12] Beketov MA, Foit K, Schaefer RB, Schriever CA, Sacchi A, Capri E (2009). SPEAR indicates pesticide effects in streams—Comparative use of species–and family-level biomonitoring data. Environ Pollut.

[13] Singer H, Jaus S, Hanke I, Lück A, Hollender J, Alder AC (2010). Determination of biocides and pesticides by on-line solid phase extraction coupled with mass spectrometry and their behaviour in wastewater and surface water. Environ Pollut.

[14] Grantham TE, Canedo-Argüelles M, Perrée I, Rieradevall M, Prat N (2012). A mesocosm approach for detecting stream invertebrate community responses to treated wastewater effluent. Environ Pollut.

[15] Abegglen C, Escher BI, Hollender J, Koepke S, Ort C, Peter A (2009). Ozonation of treated effluent—final report pilot plant Regensdorf.

[16] Bundschuh M, Pierstorf R, Schreiber WH, Schulz R (2011). Positive effects of wastewater ozonation displayed by in situ bioassays in the receiving stream. Environ Sci Technol.

[17] Underwood AJ (1994). On beyond BACI: sampling designs that might reliably detect environmental disturbances. Ecol Appl.

[18] Stewart-Oaten A, Bence JR, Osenberg CW (1992). Assessing effects of unreplicated perturbations: no simple solutions. Ecology.

[19] Dyer SD, Wang X (2002). A comparison of stream biological responses to discharge from wastewater treatment plants in high and low population density areas. Environ Toxicol Chem.

[20] Jin SR, Sang DK, Nam IC, An KG (2007). Ecological health assessments based on whole effluent toxicity tests and the index of biological integrity in temperate streams influenced by wastewater treatment plant effluents. Environ Toxicol Chem.

[21] Liess M, Schaefer RB, Schriever CA (2008). The footprint of pesticide stress in communities-species traits reveal community effects of toxicants. Sci Total Environ.

[22] AquaPlus (2009) Furtbach (ZH): biologische untersuchungen oberhalb und unterhalb der ARA Regensdorf—äusserer aspekt, pflanzlicher bewuchs, kieselalgen und zoobenthos. untersuchungen der jahre 2006, 2007 und 2008. Kurzbericht zürich: AquaPlus, commisioned by Baudirektion Kanton Zürich, AWEL, Abteilung Gewässerschutz

[23] BUWAL (2005) Methoden zur untersuchung und Beurteilung der Fliessgewässer. Makrozoobenthos Stufe F (flächendeckend)

[24] Liess M, Von Der Ohe PC (2005). Analyzing effects of pesticides on invertebrate communities in streams. Environ Toxicol Chem.

[25] Wogram J, Liess M (2001). Rank ordering of macroinvertebrate species sensitivity to toxic compounds by comparison with that of *Daphnia magna*. Bull Environ Contam Toxicol.

[26] Rubach MN, Baird DJ, Van Den Brink P (2010). A new method for ranking mode-specific sensitivity of freshwater arthropods to insecticides and its relationship to biological traits. Environ Toxicol Chem.

[27] Schaefer RB, Caquet T, Siimes K, Mueller R, Lagadic L, Liess M (2007). Effects of pesticides on community structure and ecosystem functions in agricultural streams of three biogeographical regions in Europe. Sci Total Environ.

[28] Cumming G (2014). The new statistics: why and how. Psychol Sci.

[29] Halsey LG, Curran-Everett D, Vowler SL, Drummond GB (2015). The fickle P value generates irreproducible results. Nat Methods.

